# Exploring the association of dietary patterns with the risk of hypertension using principal balances analysis and principal component analysis

**DOI:** 10.1017/S136898002200091X

**Published:** 2022-04-13

**Authors:** Junkang Zhao, Wenjing Guo, Juping Wang, Tong Wang

**Affiliations:** Department of Health Statistics, School of Public Health, Shanxi Medical University, 56 Xinjiannanlu Street, Taiyuan 030001, China

**Keywords:** Dietary patterns, Compositional data, Principal balance, Principal component analysis, Coarse cereals, Hypertension, China health and nutrition survey

## Abstract

**Objective:**

In the field of nutritional epidemiology, principal component analysis (PCA) has been used extensively in identifying dietary patterns. Recently, compositional data analysis (CoDA) has emerged as an alternative approach for obtaining dietary patterns. We aimed to directly compare and evaluate the ability of PCA and principal balances analysis (PBA), a data-driven method in CoDA, in identifying dietary patterns and their associations with the risk of hypertension.

**Design:**

Cohort study. A 24-h dietary recall questionnaire was used to collect dietary data. Multivariate logistic regression analysis was used to analyse the association between dietary patterns and hypertension.

**Setting:**

2004 and 2009 China Health and Nutrition Survey.

**Participants:**

A total of 3892 study participants aged 18–60 years were included as the subjects.

**Results:**

PCA and PBA identified five patterns each. PCA patterns comprised a linear combination of all food groups, whereas PBA patterns included several food groups with zero loadings. The coarse cereals pattern identified by PBA was inversely associated with hypertension risk (highest quintile: OR = 0·74 (95 % CI 0·57, 0·95); *P*_for trend_ = 0·037). None of the five PCA patterns was associated with hypertension. Compared with the PCA patterns, the PBA patterns were clearly interpretable and accounted for a higher percentage of variance in food intake.

**Conclusions:**

Findings showed that PBA might be an appropriate and promising approach in dietary pattern analysis. Higher adherence to the coarse cereals dietary pattern was associated with a lower risk of hypertension. Nevertheless, the advantages of PBA over PCA should be confirmed in future studies.

Hypertension is a growing public health concern worldwide due to its major role in the morbidity and mortality of chronic diseases. According to reports, the number of adults aged >20 years with high blood pressure is expected to rise from 920 million in 2000 to 1·56 billion in 2025^(1)^. In response to these findings, numerous studies have demonstrated that diet plays an important role in effectively preventing hypertension, lowering the blood pressure of hypertensive patients and improving the effect of antihypertensive therapy^(2)^.

Studies on diet and blood pressure have increasingly focused on dietary patterns, such as the Dietary Approaches to Stop Hypertension diet^(3)^, Mediterranean dietary pattern^(4)^ and Western dietary pattern^(5)^, rather than the traditional single-nutrient approach, due to the complex interactions and correlations among nutrients and food components^(6)^. However, statistical methods for identifying these dietary patterns vary. The *a priori* and *a posteriori* methods are the most popular approaches used for extracting dietary patterns in observational studies^(7)^. The *a priori* method is mainly based on prior knowledge or theories regarding a healthy diet, such as the Dietary Approaches to Stop Hypertension diet scores^(8)^ and the Healthy Eating Index^(9)^. However, these scores only focus on particular dietary aspects and do not consider correlations between nutrients^(10)^. Conversely, the *a posteriori* method is data driven, and dietary patterns are derived from statistical dimension reduction techniques^(7)^. Principal component analysis (PCA) is the most frequently used data-driven method. In PCA, the original food variables are replaced with new variables (factors or components). PCA, however, is not entirely data driven due to the subjectivity in the selection of rotation methods, the threshold value of foods’ factor loadings and the foods for labelling^(11)^. Furthermore, qualitative data interpretation is challenging, because the components of the analysis include all food groups^(11)^. As the amount of food intake is relatively constant, changes in the intake of some foods will lead to a corresponding decrease or increase in the intake of other foods; that is, foods are co-dependent during intake, implying the substitutional nature of dietary data^(12)^. Therefore, dietary intake can also be considered as compositional data^(12)^. PCA is commonly used to investigate different food combinations, providing information on which foods in the dietary pattern are consumed more frequently; however, the compositional nature of dietary data is not always handled appropriately.

Compositional data are positive values representing some parts of a whole, which can be either varied (e.g. total food intake) or fixed (e.g. 24 h/d)^(13)^. Hence, the relative importance of the parts is the main concern. These properties create challenges for standard statistical methods conceived for unconstrained variables^(13)^. Compositional data analysis (CoDA) is a standard family of log-ratio methods for analysing the relative importance of variables and has great potential in the field of nutritional epidemiology^(14)^. In nutrients research, Maria Léa Corrêa Leite used isometric log-ratio transformation and sequential binary partition to investigate the relationships between macronutrient composition and diseases and demonstrated their potential advantages over the usual analytical methods^(15,16)^. Subsequently, the author extended the use of CoDA to micronutrient composition (vitamins and minerals) to evaluate and interpret the relative roles of different dietary components within a holistic overview of a diet^(17)^. In addition, similar to PCA, CoDA can also reduce the dimension of compositional data^(18)^. Currently, several methods have been proposed for extracting dietary patterns using CoDA, including compositional PCA, balances analysis and principal balances analysis (PBA), to emphasise the relative importance and substitution effects of different food groups^(11,12)^. Using CoDA, one dietary pattern is seen as a trade-off between the increased intake of some foods and a decreased intake of others. Among the three CoDA methods, compositional PCA can be considered as a standard PCA based on the data being appropriately transformed^(12)^; hence, it has the same problem as that in PCA, that is, each principal component coordinate generally involves all food groups, which makes it difficult to explain. Balances are constructed based on a sequential binary partition, which is investigator-driven and involves only some (not all) food groups that have a simple interpretation^(19)^. However, the food groups in balances are selected by investigators or based on research questions of interest that introduce a degree of subjectivity. In addition, a large percentage of total variance cannot concentrate on a few balances. PBA, a recently developed method, has received increasing attention because it concentrates on most of the variance in the sample of a few variables like compositional PCA, and these variables have the advantage of easy interpretability like balances^(11,12,18)^. Thus, PBA can be viewed as equivalent to balances, which are constructed through data-driven methods and focus on variation in the food groups. However, to the best of our knowledge, the association between CoDA-identified dietary patterns and health outcomes has not been identified. Furthermore, no direct comparison of CoDA methods and traditional dietary pattern analysis methods has been performed.

Therefore, the primary objectives of this study were to (1) directly compare dietary patterns identified by PCA and PBA and (2) investigate the associations of these dietary patterns with the risk of hypertension.

## Methods

### Study design and population

Participants of the China Health and Nutrition Survey (CHNS), which is an ongoing large-scale, prospective cohort survey initiated in 1989, were included in this study. Multi-stage random cluster sampling was performed to select the study participants from nine provinces with varied data on demography, geography, economic development and public resources. The CHNS is described in detail elsewhere^(20)^.

**Fig. 1:**
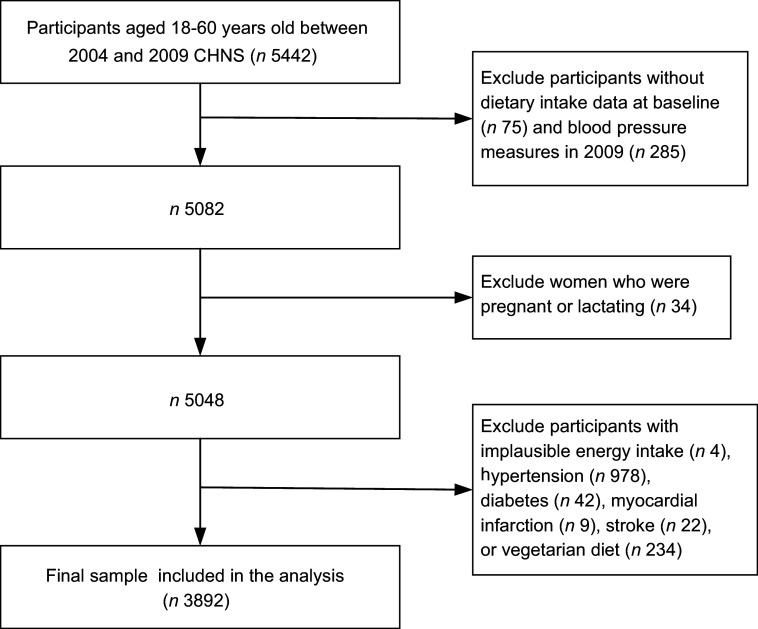
Flow chart of the analysis sample

Data from surveys conducted at both baseline (2004) and follow-up (2009) were used, because the way the overall dietary intake remained relatively stable between 1991 and 2009^(21)^. In total, 5442 individuals aged 18–60 years who participated at baseline were included. Among these adults, pregnant or lactating mothers (*n* 34) and participants with implausible energy intake (*n* 4), hypertension (*n* 978), diabetes (*n* 42), myocardial infarction (*n* 9), stroke (*n* 22), vegetarian diets (*n* 234), missing dietary intake values at baseline (*n* 75) and missing blood pressure values in 2009 (*n* 285) were excluded. Overall, the data of 3892 participants were retained for analysis (Fig. 1).

### Dietary assessment

Both individual-level and household-level diet data of the CHNS were collected by well-trained research staff within 3 d. Individual dietary intake data were collected using a 24-h recall questionnaire. To obtain average intake data for each participant, they were asked to report the type and quantity of foods and beverages consumed during a 24-h period on three consecutive days. The procedure for dietary measurement has been described in detail elsewhere^(22)^. Based on nutritional components or culinary uses, the individual food items were combined into twenty food groups (Additional File 1).

### Blood pressure and hypertension

Blood pressure was measured thrice using a mercury sphygmomanometer with the appropriate cuff size for each participant, who was seated during the measurement. The participants were asked to rest for at least 5 min before blood pressure measurement. To confirm hypertension, the average blood pressure from triplicate independent measurements on the same arm was considered. The outcome variable in our study was incidence of new-onset hypertension between 2004 and 2009. Participants with an average systolic blood pressure (SBP) of ≥140 mmHg and/or diastolic blood pressure (DBP) of ≥90 mmHg, those who were diagnosed with hypertension by a physician, or those using anti-hypertensive medications between 2004 and 2009 were considered to have hypertension.

### Other variables

Socio-demographic characteristics (age, sex, residence (urban, rural), region (north, south), marital status (unmarried, married, divorced, widowed or separated), education level (primary school or less, middle school, high school or more), annual per capita income) and lifestyle factors (smoking status (never, former, current), alcohol consumption habits (current, never), sleep duration, physical activity time, sedentary time) were obtained using a baseline questionnaire. Height and weight were measured using calibrated equipment under standardised conditions. The BMI was calculated as the quotient of body weight in kilograms divided by the square of height in meters (kg/m^2^).

### Statistical analysis

#### Dietary analysis

The dietary patterns were obtained using PCA and PBA of data from the 3892 participants and twenty food groups. Using the 24-h dietary recall questionnaire within a 3-d period, a large proportion of the participants were found to be non-consumers of many food groups. For PCA, individual food groups with <25 % consumers were categorised as binary variables (non-consumers *v*. consumers); food groups with >25 % consumers were categorised as three-level variables (non-consumers and consumers with dietary intake above/below median). These categories were used to construct a polychoric correlation matrix. The food groups with <5 % consumers were excluded. Finally, nineteen food groups were used in the PCA correlation matrix to produce principal components (PC). The first PC could maximise the variation in dietary data, and each subsequent component maximised the remaining variance, while ensuring that it was independent of the previous components. PC were rotated using the varimax orthogonal rotation technique to allow for greater interpretability. Results of the Kaiser–Meyer–Olkin test and Bartlett’s test of sphericity suggested that the present food intake data were suitable for PCA. For each PC, food groups with factor loadings of |≥0·3| were retained for the labelling of patterns. The following conditions were considered in determining the number of key dietary patterns: eigenvalues >1·0, the break in the scree plot and the interpretability of the identified patterns. The PCA of categorical food groups was conducted using SAS version 9.4 (SAS).

In CoDA, the isometric log-ratio transformation is seemed as the most appropriate method for transforming compositional data to construct new variables in real space, so that standard multivariate statistics can be used directly^(23)^. For dietary patterns analysis, PBA is an recommended data-driven procedures based on sequential binary partition to compute the isometric log-ratios, which is called principal balances (PB)^(18)^. The general expression can be expressed as

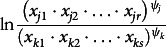

where r and s, respectively, represent the number of parts in the numerator and denominator at each order of partition. All these non-zero exponents 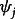 and 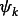 of the food groups characterise the PB that measure the relative weight of one group of foods against the other foods. Similar to PCA, PBA can concentrate most of the sample variances to few PB. The first PB has the largest sample variance; the second PB has the second greatest variance, and so on. However, in contrast to PCA, PB can also be regarded as a linear combination of the logarithms of a smaller number of food groups; this makes the interpretation and labelling of patterns easier. While computing log-ratios, zero values cannot exist in all food groups. Participants with absolute zeros were excluded; these values resulted from non-consumption of meat, eggs, milk and seafood (i.e. vegans or vegetarians). Other zeros, known as rounded zeros, are considered as existing, but were missing because the 3-d 24-h dietary recall questionnaire failed to capture less frequently consumed foods. These zero values are commonly imputed through the modified expectation–maximisation algorithm with a lower detection limit^(24)^.

The variations in food groups can be deconstructed into variations explained by the PC or PB. They can be included as independent variables in the regression model. Quartiles were constructed for each PCA or PBA dietary pattern and used to assess the relationship between dietary patterns and the risk of hypertension.

#### Descriptive analysis and modelling

The continuous variables are presented as means with SD; the categorical variables are presented as frequencies and proportions. The differences in baseline characteristics between the participants with and without hypertension were examined using two independent sample *t*-tests or *χ* ^2^ tests. The associations of dietary patterns with the risk of hypertension were examined using multivariable logistic regression models. One set of three models was used: model 1, adjusted for age and sex; model 2, additionally adjusted for residence, region, marital status, education level, per capita annual family income, smoking status, drinking habits, sleep duration, physical activity time, sedentary time and total energy and model 3, adjusted for BMI, SBP at baseline, DBP at baseline and model 2 variables.

The trend of associations was estimated using quartiles of PC or PB scores as continuous variables. The missing data in the covariates were assumed to be missing at random^(25)^. Therefore, multiple imputations with the chained equation method were used to impute missing data. The selection of linear or logistic regression modelling of the missing data depended on the type of covariates. Ten imputed data sets were created, and their analysis results were pooled to obtain the final results. In addition, BMI may be a potential mediator between diet and hypertension^(26,27)^. Therefore, we performed a mediation analysis to test whether BMI has a mediation effect on the relationships between dietary patterns and the risk of hypertension.

A two-sided *P* < 0·05 was considered statistically significant. The predictive accuracy of the models was assessed using Akaike’s information criterion. All analyses except PCA were performed using R statistical software, version 4.0.3. For PBA, the R packages ‘coda.base’ and ‘robCompositions’ were used. For multiple imputations, the R package ‘MICE’ was used.

## Results

### Characteristics of study participants

The baseline participants’ characteristics and a comparison of participants with and without hypertension are illustrated in Table 1. The mean age at baseline was 42·26 (sd, 10·02 years) years; 1755 (45·09 %) participants were men and 2137 (54·91 %) were women. Univariate analysis revealed that, compared with those without hypertension, participants with hypertension were predominantly older, males, living in the northern region, having an education level of primary school or less, married, divorced, widowed or separated, former or current smokers, alcohol consumers, having a higher BMI and having higher baseline SBP and baseline DBP. Among all the participants, 1119 (28·75 %) had at least one missing value in the covariates.

**Table 1:** Baseline characteristics of 3892 study participants of the CHNS

Characteristic			Hypertension				*P* value*
	All participants (*n* 3892)		No (*n* 3188)		Yes (*n* 704)		
	Mean, *n*	sd, %	Mean, *n*	sd, %	Mean, *n*	sd, %	
Age (year)	42·26	10·02	41·72	10·08	45·83	9·03	<0·001
Men	1755	45·09	1391	43·63	364	51·70	<0·001
Urban residence	1191	30·60	995	31·21	196	27·84	0·079
Northern region	1606	41·26	1278	40·09	328	46·59	0·002
Educational level†							0·001
Primary school or less	1482	38·08	1177	36·92	305	43·32	
Middle school	1438	36·95	1183	37·11	255	36·22	
High school or more	933	23·97	796	24·97	137	19·46	
Unknown	39	1·00	32	1·00	7	0·99	
Marriage status†							0·001
Unmarried	236	6·06	210	6·59	26	3·69	
Married	3481	89·44	2848	89·36	633	89·91	
Divorced, widowed or separated	129	3·31	94	2·95	35	4·97	
Unknown	46	1·18	36	1·13	10	1·42	
Smoking status†							0·029
Never	2643	67·91	2194	68·82	449	63·78	
Former	95	2·44	76	2·38	19	2·70	
Current	1112	28·57	883	27·70	229	32·53	
Unknown	42	1·08	35	1·10	7	0·99	
Drinking status†							<0·001
Never	2715	69·76	2262	70·95	453	64·35	
Current	1133	29·11	889	27·89	244	34·66	
Unknown	44	1·13	37	1·16	7	0·99	
Annual/capita income†	6803·17	8787·78	6841·60	8727·71	6621·41	9072·76	0·601
BMI, kg/m^2^ †	22·79	3·04	22·55	2·92	23·92	3·31	<0·001
Sleep duration (h/d)†	8·12	1·15	8·13	1·13	8·05	1·21	0·084
Physical activity time (h/d)†	0·96	2·56	0·94	2·49	1·01	2·81	0·690
Sedentary time (h/d)†	5·55	4·03	5·56	4·02	5·48	4·06	0·652
Total energy (kJ/d)†	2281·98	651·14	2275·68	650·52	2310·49	653·64	0·199
SBP at baseline†	114·63	10·99	113·61	10·97	119·23	9·83	<0·001
DBP at baseline†	74·99	7·91	74·34	7·93	77·91	7·12	<0·001

CHNS, China health and nutrition survey; SBP, systolic blood pressure; DBP, diastolic blood pressure.

* *P* values were obtained using the two independent sample *t*-tests or *χ* ^2^ tests, where appropriate, and the category ‘unknown’ was not used for *χ* ^2^ tests.

† Missing data: 867 for annual per capita income, 257 for BMI, 125 for sleep duration, 52 for physical activity time, 55 for sedentary time, 2 for total energy, 216 for SBP at baseline and 216 for DBP at baseline.

### Dietary patterns based on principal component analysis and principal balance

**Fig. 2:**
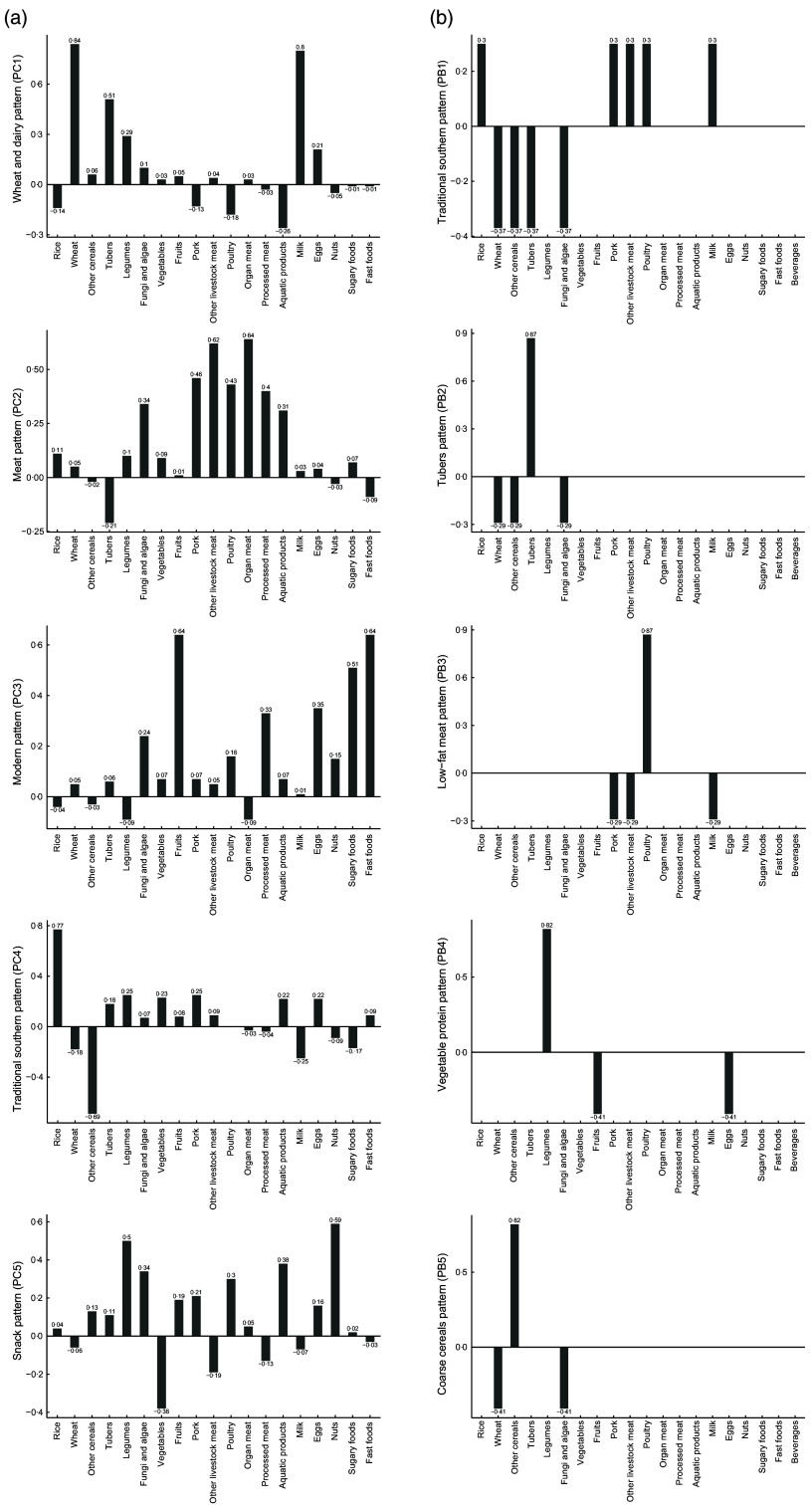
Factor loadings and exponents for dietary patterns derived using principal component analysis (a) and principal balance analysis (b), respectively. PC, principal component. PB, principal balance

PCA extracted five dietary patterns; five dietary patterns were retained for PBA, for comparison purposes. The loadings or exponents of the five food patterns are presented in Fig. 2. According to the extracted loadings (values >0·3) using PCA: PC1 (the wheat and dairy pattern) was characterised by a high intake of wheat, tubers and milk; PC2 (the meat pattern) was characterised by a high intake of pork, other livestock meat, poultry, organ meat, processed meat, aquatic products and fungi and algae; PC3 (the modern pattern) was characterised by a high intake of fruits, processed meat, eggs, sugary foods and fast foods; PC4 (the traditional southern pattern) was characterised by a high intake of rice and a low intake of other cereals and PC5 (the snack pattern) was characterised by the high intake of nuts, legumes, aquatic food, fungi and algae, poultry and a low intake of vegetables. For PB, the food groups with non-zero exponents were used for labelling. PB1 (the traditional southern pattern) represented the comparison between rice, pork, other livestock meat, poultry and milk *v*. wheat, other cereals, tubers and fungi and algae. PB2 (the tubers pattern) represented the comparison between tubers *v*. wheat, other cereals and fungi and algae. PB3 (the low-fat meat pattern) represented the comparison between poultry *v*. pork, other livestock meat and milk. PB4 (the vegetable protein pattern) represented the comparison between legumes *v*. fruits and eggs. PB5 (the coarse cereals pattern) represented the comparison between other cereals *v*. wheat and fungi and algae.

The percentage of variations accounted for by PC or PB are shown in Table 2. The first five PC explained 39·99 % of the food intake variation, whereas the first five PB explained 51·35 % of the food intake variation (Table 2).

**Table 2:** The percentages of variation in all food groups explained using the principal component analysis and principal balances analysis

Dietary pattern	Variance explained (%)	
	Principal component analysis	Principal balances analysis
PC/PB 1	12·22	17·02
PC/PB 2	9·93	9·56
PC/PB 3	6·33	8·55
PC/PB 4	6·12	8·49
PC/PB 5	5·40	7·74
Total	39·99	51·35

PC, principal component; PB, principal balance.

### Dietary patterns and hypertension

During the 5-year follow-up period, 704 participants developed hypertension. The incidence of hypertension was 18·09 %. The five dietary patterns derived from PCA or PBA were used as predictors of hypertension. After adjustment for all potential confounders (in Model 3), no PCA dietary pattern was independently associated with the risk of hypertension (Table 3). Only the coarse cereals pattern of the PBA was inversely associated with hypertension (adjusted OR for quintile 4 *v*. quintile 1: 0·74; 95 % CI (0·57, 0·95); *P*_trend_ = 0·037); that is, participants who had a higher coarse cereals pattern score had a lower risk of hypertension (Table 3). Moreover, the above association was not changed by other adjustments for hypertension. In a mediation analysis, we did not find a significant mediation effect of BMI on dietary patterns–hypertension relationships. Hence, the association between the coarse grain pattern and the risk of hypertension was independent of the potentially mediating factor, BMI (data not shown). Regression analysis revealed that the Akaike’s information criterion of the model using PBA was similar to that of the model using PCA (3391·54 *v*. 3404·21, respectively), indicating that the predictive accuracy of the model using PBA was higher.

**Table 3:** The association of the dietary patterns with the risk of hypertension

	Q1	Q2		Q3		Q4		*P* for trend
		OR	95 % CI	OR	95 % CI	OR	95 % CI	
Principal component analysis								
PC1: Wheat and dairy pattern								
Model 1	Ref.	1·20	0·94, 1·54	1·15	0·90, 1·46	1·23	0·96, 1·58	0·111
Model 2	Ref.	1·14	0·88, 1·46	1·08	0·84, 1·38	1·09	0·84, 1·41	0·626
Model 3	Ref.	1·02	0·79, 1·33	1·07	0·82, 1·38	1·02	0·78, 1·33	0·827
PC2: Meat pattern								
Model 1	Ref.	1·06	0·84, 1·35	0·84	0·65, 1·08	1·02	0·80, 1·30	0·788
Model 2	Ref.	1·14	0·90, 1·46	0·95	0·73, 1·23	1·28	0·98, 1·66	0·145
Model 3	Ref.	1·09	0·85, 1·39	0·87	0·66, 1·13	1·15	0·88, 1·50	0·616
PC3: Modern pattern								
Model 1	Ref.	0·87	0·68, 1·11	1·02	0·81, 1·30	0·82	0·64, 1·04	0·278
Model 2	Ref.	0·87	0·68, 1·12	0·97	0·76, 1·24	0·79	0·61, 1·03	0·186
Model 3	Ref.	0·89	0·69, 1·15	0·98	0·76, 1·26	0·82	0·62, 1·07	0·282
PC4: Traditional southern pattern								
Model 1	Ref.	1·05	0·82, 1·34	1·04	0·82, 1·33	1·09	0·85, 1·40	0·667
Model 2	Ref.	1·28	0·98, 1·64	1·27	0·98, 1·64	1·29	0·99, 1·68	0·080
Model 3	Ref.	1·30	0·10, 1·70	1·27	0·97, 1·65	1·29	0·99, 1·69	0·091
PC5: Snack pattern								
Model 1	Ref.	1·14	0·90, 1·46	1·00	0·77, 1·29	1·02	0·79, 1·32	0·524
Model 2	Ref.	1·20	0·94, 1·53	1·06	0·81, 1·38	1·13	0·87, 1·47	0·810
Model 3	Ref.	1·10	0·85, 1·41	0·98	0·75, 1·29	1·03	0·78, 1·35	0·408
Principal balances								
PB1: Traditional southern pattern								
Model 1	Ref.	0·70	0·54, 0·91	0·79	0·59, 1·06	1·07	0·79, 1·46	0·599
Model 2	Ref.	0·72	0·55, 0·94	0·82	0·61, 1·11	1·12	0·82, 1·53	0·434
Model 3	Ref.	0·74	0·56, 0·97	0·82	0·60, 1·11	1·14	0·83, 1·57	0·426
PB2: Tubers pattern								
Model 1	Ref.	0·84	0·65, 1·10	0·82	0·64, 1·05	1·07	0·84, 1·36	0·590
Model 2	Ref.	0·84	0·64, 1·09	0·83	0·64, 1·06	1·06	0·83, 1·35	0·671
Model 3	Ref.	0·82	0·62, 1·08	0·82	0·64, 1·06	1·15	0·90, 1·48	0·330
PB 3: Low-fat meat pattern								
Model 1	Ref.	0·94	0·74, 1·20	0·90	0·70, 1·15	0·89	0·70, 1·13	0·366
Model 2	Ref.	0·93	0·73, 1·19	0·90	0·70, 1·15	0·90	0·70, 1·15	0·410
Model 3	Ref.	0·93	0·72, 1·19	0·90	0·69, 1·15	0·88	0·68, 1·13	0·331
PB 4: Vegetable protein pattern								
Model 1	Ref.	0·98	0·77, 1·24	1·07	0·84, 1·36	1·18	0·93, 1·50	0·164
Model 2	Ref.	0·96	0·75, 1·23	1·09	0·85, 1·38	1·16	0·91, 1·47	0·208
Model 3	Ref.	1·04	0·81, 1·34	1·06	0·83, 1·36	1·13	0·88, 1·45	0·326
PB 5: Coarse cereals pattern								
Model 1	Ref.	0·73	0·57, 0·93	0·79	0·61, 1·02	0·75	0·58, 0·96	0·043
Model 2	Ref.	0·72	0·57, 0·92	0·77	0·59, 1·00	0·74	0·58, 0·95	0·036
Model 3	Ref.	0·69	0·54, 0·89	0·74	0·57, 0·97	0·74	0·57, 0·95	0·037

PC, principal component; PB, principal balance.

Model 1 was adjusted for sex and age.

Model 2 was additionally adjusted for residence, region, marital status, education level, per capita annual family income, smoking status, drinking habits, sleep duration, physical activity time, sedentary time and total energy.

Model 3 was additionally adjusted for BMI, SBP at baseline and DBP at baseline.

*P*_for trend_ was calculated by including the tertiles of the factor scores as continuous variables in the models.

## Discussion

This study provides evidence of the association between dietary patterns and hypertension using PCA and PBA. To the best of our knowledge, this is the first study to compare the dietary patterns derived from both methods and assess the association between dietary patterns and hypertension. We identified five dietary patterns for each method that showed similar accuracy in predicting hypertension. However, by comparing the variance explained by the two methods, it was observed that the variations of food groups captured by the first five PB in PBA were higher than those captured by the PC derived from PCA. Among the five dietary patterns identified by both methods, none derived from PCA was significantly associated with hypertension; however, PBA identified the coarse cereals pattern as inversely related to the risk of hypertension, independent of other covariates. Therefore, it could be interpreted that increasing the intake of other cereals (maize, barley, and millet) by a proportion while decreasing the intake of wheat and fungi and algae by the same proportion is associated with a lower hypertension risk.

Coarse food grains are the traditional grain source in Chinese diets. They mainly include grains or beans other than rice and wheat grains, which are similar to the components of the coarse cereals pattern^(28)^. Indeed, millet, corn, oats, adlay, buckwheat and their products, which are the same as the food items in the other cereals food group in our study, are the main forms of coarse food grains in China^(28)^. Compared with whole grains, coarse food grains contain similar nutrients, such as vitamin B, fibre and several trace minerals. In addition, they are cheaper and more available. These factors make them a proxy to whole grains in China^(29)^. To the best of our knowledge, no study has found a relationship between the coarse cereals pattern identified by dietary pattern analyses and hypertension. However, several studies have suggested that coarse food grains or whole grains are beneficial for lowering blood pressure. In China, a cross-sectional study of 104 men and women aged 18–35 years indicated that the frequent consumption of whole grains can help lower both SBP and DBP^(29)^. This was consistent in a study of Chinese people aged 30–79 years^(30)^. In Japan, a prospective study of an Asian population reported that individuals who consumed whole grains frequently had a 64-percent decreased risk of hypertension, compared with those who never consumed whole grains^(31)^. Similar findings have been observed in western populations: a meta-analysis of cohort studies performed in the USA reported that the intake of whole grains was inversely associated with the risk of hypertension^(32)^. According to the most recent cohort study involving 3121 participants and multiple follow-up examinations, the consumption of ≥48 g/d of whole grains resulted in a smaller average increase in SBP every 4 years, compared with the consumption of <8 g/d of whole grains (0·2 mmHg *v*. 1·4 mmHg)^(33)^. Wheat products are an important component of refined grains, which comprise the majority of grains consumed in China^(34)^. Some studies have supported our findings. For example, one cross-sectional study from Tehran indicated that the frequent intake of refined grain products, notably noodles, could increase the risk of hypertension by 69 %^(35)^. One cohort study of Korean women aged 40–69 years found that consuming five or more servings of noodles/week could increase the risk of hypertension by 1·31-fold, compared with never consuming noodles^(36)^. Fungi and algae are healthy foods that prevent hypertension^(37,38)^. However, this study showed an inverse association of coarse cereals patterns (consisting of a high consumption of other cereals and a low intake of wheat and fungi and algae) with the incidence of hypertension. This is because our study focused on the importance of the overall dietary pattern. The adverse effects of a low intake of fungi and algae on blood pressure may be offset by a higher intake of coarse food grains and a lower intake of wheat. In 2015, the United States Dietary Guideline Advisory Committee also recommended that whole grains should be used as a substitute for most refined grains to improve dietary quality^(39)^. The results of these studies are in line with our findings using PBA.

The specific mechanisms underlying the benefits of the coarse cereals pattern on hypertension are still unclear. However, several potential mechanisms have been reported. Recently, an animal experiment involving hypertensive mice showed that a fibre-rich diet could cause changes in the gut microbiota that help in the prevention of CVD and down-regulation of the gene network of the renal renin-angiotensin-aldosterone system; this is helpful in lowering blood pressure^(40)^. A meta-analysis reported that a *β*-glucan-rich diet effectively reduced the SBP and DBP by 7·5 mmHg and 5·5 mmHg, respectively^(41)^. An untargeted metabolomic and lipidomic profiling study found that sphingolipid ceramides, triacylglycerols, phosphatidylcholines and phosphatidylethanolamine may create a link between consumption of coarse food grains and SBP or/and DBP^(42)^. A randomised trial with a parallel design demonstrated that the intake of whole grains may affect blood pressure by increasing vascular reactivity^(43)^. The potential beneficial effects of coarse food grains on blood pressure control may also be mediated by folates and vitamin B_6_, which are abundant in coarse food grains^(44,45)^. A randomised controlled trial showed that long-term homocysteine-lowering treatment with folic acid plus vitamin B_6_ is associated with reduced blood pressure^(46)^. Other studies have also reported that increasing the intake of vitamin B_6_ and folic acid can effectively lower plasma homocysteine levels^(47)^, thus preventing vascular injury^(48)^ and reducing the risk of H-type hypertension (combination of primary hypertension and hyperhomocysteinaemia), which is very common in the Chinese population^(49)^. Other nutrients in whole grains, such as Mg, potassium, Se and Zn; antioxidants and polyphenols, which exist in relatively low levels in refined grains, may help in reducing blood pressure^(50)^. Additionally, the effect of increased blood pressure caused by the intake of refined grains represented by noodle dishes may be caused by their high Na content^(36)^.

The advantage of PBA can be shown by describing the methodological differences between the two methods. First, their procedures for creating patterns are different. PCA creates patterns based on the correlation of all the real food groups. Individual dietary patterns can be represented by derived PC scores, which are a linear combination of all food groups and are usually interpreted as the greater consumption of some food groups. PBA produces patterns by continuously partitioning the parts of compositional dietary data that are expressed as a percentage of each food group. Each pattern is interpreted as the trade-off between consuming more of the food groups in the numerator and less of the food groups in the denominator. As shown in Fig. 2, the factor loadings of the retained food groups in PC1, PC2 and PC3 were all positive; however, those of PC4 and PC5 were not. These results were not constant; negative loadings also appeared in PC1 and PC2 when 0·2 was selected as the threshold value for the food groups retained in the patterns, instead of 0·3. However, subsequent challenges will be posed by the increasing complexity of labelling and interpretation of PC. Thus, this shows that PCA focuses more on different food combinations rather than on substitution effects. However, the exponents of the five patterns identified by PBA were all positive and negative. Therefore, in addition to focusing on different food combinations, the interpretation of each pattern is also characterised by the substitution effects of the food group(s). Another difference is the manner of simplifying the interpretation of patterns. PBA automatically produces sparse patterns involving a smaller number of food groups, which are simpler to interpret. However, the non-zero loadings of all the food groups in PCA complicate the characterisation of patterns. To provide a simple structure of PC and produce patterns that are easier to explain using PCA, factor rotation (orthogonal or oblique rotation) is usually performed. Our study used orthogonal rotation (which is the most commonly used method) to provide optimal uncorrelated PC. However, the rotation method choice is subjective and may influence the final results.

PBA also has some limitations. The main limitation is the absence of standard and objective approaches to determine the number of patterns to be retained. To facilitate the comparison between methods, the same number of patterns was retained for both PBA and PCA during this study. Although a combination of variances and interpretability considerations (the ability to interpret the retained patterns) to decide the number of retained patterns, this is a completely subjective decision. In addition, PBA may not always derive clearly defined and sensible patterns that can be straightforwardly translated into dietary advice. For example, the intake of fungi and algae was positively associated with the risk of hypertension in the coarse cereals pattern in our study, which is not consistent with previous studies^(37,38)^. This is because, like other data-driven methods, PBA focuses only on explaining the variance in food intake and does not make use of prior knowledge or suspected diet-disease relationships. Another major concern is the large number of zeros undermining the application of the compositional approach. The more detailed the food groups, the more likely is the presence of zeros. Therefore, before applying log-ratios transformation, zeros that are typically present in the food groups must be considered. Currently, the rounded zeros can be imputed in several ways, such as through the use of parametric or non-parametric approaches, to preserve the covariance structure of original variables as much as possible. However, there is currently no well-founded general approach for rounded zeros, and further improvements are required.

The present study had several strengths, including the longitudinal study design, larger sample size, use of 3-d dietary records for dietary assessment, detailed information about lifestyle factors, high quality of follow-up, various evaluation indices for comparison and the use of compositional data analysis methods, reflecting the recent developments in statistical approaches for dietary data in the identification of dietary patterns. However, this study also had some limitations. First, data regarding the habitual intake of episodically consumed foods and seasonal diet variability were difficult to obtain through a 3-d dietary survey. Second, the consolidation of food items into food groups and labelling for dietary patterns were subjective. Third, only Chinese participants were included; this may limit the generalisability of the findings to other populations. Fourth, there was potential residual confounding due to the observational nature of the study.

## Conclusions

In summary, PCA and PBA can both be regarded as statistical methods that produce new variables representing dietary patterns and explain the total sample variation. However, this study showed that the dietary pattern derived from PBA is more suitable for the common practice of dietary advice and intuitive concept of dietary patterns. Furthermore, an inverse association was found between the coarse cereals pattern derived from PBA and the risk of hypertension; this was not revealed when PCA was used. These findings suggest that PBA may be a more appropriate statistical method in dietary pattern analyses, which considers the compositional nature of the diet and can be considered as an alternative for extracting dietary patterns that can complement traditional methods. However, the advantages of PBA must be validated in future studies conducted in other settings, including different health outcomes and population groups.

## Supplementary material

For supplementary material accompanying this paper visit https://doi.org/ 10.1017/S136898002200091X. click here to view supplementary material
