# Supplementary material for: Exploring the association of dietary patterns with the risk of hypertension using principal balances analysis and principal component analysis
Source: Public Health Nutr. 2022 Apr 13;26(1):160–70. doi: 10.1017/S136898002200091X (PMC11077459; doi:10.1017/S136898002200091X)
Supplement: Supplementary file 1 [file S136898002200091Xsup001.docx]

**Additional file 1:**

**Table S1.** Examples of food items for each food group

| **Food groups** | **Examples of food items** |
| --- | --- |
| Rice | Round-grained rice, long-grained rice, glutinous rice |
| Wheat | Wheat bun, wheat noodles |
| Other cereals | Maize, barley, millet |
| Tubers | Potato, sweet potato |
| Legumes | Soyabeans, and products |
| Fungi and algae | Mushroom, kelp, laver |
| Vegetables | Cabbage, eggplant, carrot, pepper, lettuce, rape, tomato, cauliflower |
| Fruits | Apple, pear, peach, date, grape, watermelon, orange, other fruit |
| Pork | Pork and pork products |
| Other livestock meat | Beef, game, lamb, meat products |
| Poultry | Chicken, duck, goose |
| Organ meat | Organ meat |
| Processed meat | Sausages, ham, luncheon meat, dried meat, smoked meat |
| Aquatic products | Fish, shrimp, crab, shellfish |
| Milk | Milk and products |
| Eggs | Eggs |
| Nuts | Nuts |
| Sugary foods | Jelly, jam, chocolate, honey, sugar, candies |
| Fast foods | Convenience food, hamburger, pizza, sandwich, French fries |
| Beverages | Fruit or flavored drinks, fruit juice, soft drink |
